# Neuroevolution-Based Adaptive Antenna Array Beamforming Scheme to Improve the V2V Communication Performance at Intersections

**DOI:** 10.3390/s21092956

**Published:** 2021-04-23

**Authors:** Hojin Kang Kim, Raimundo Becerra, Sandy Bolufé, Cesar A. Azurdia-Meza, Samuel Montejo-Sánchez, David Zabala-Blanco

**Affiliations:** 1 Department of Electrical Engineering, Universidad de Chile, Santiago 8370451, Chile; ho.kang.k@ing.uchile.cl (H.K.K.); raimundo.becerra@ing.uchile.cl (R.B.); sbolufe@ing.uchile.cl (S.B.); 2 Programa Institucional de Fomento a la I+D+i, Universidad Tecnológica Metropolitana, Santiago 8940577, Chile; smontejo@utem.cl; 3 Centro de Investigación de Estudios Avanzados del Maule (CIEAM), Vicerrectoría de Investigación y Postgrado, Universidad Católica del Maule, Talca 3480112, Chile; dzabala@ucm.cl; 4 Department of Computer Science and Industry, Faculty of Engineering Science, Universidad Católica del Maule, Talca 3480112, Chile

**Keywords:** antenna array, genetic algorithm (GA), intelligent transport systems (ITS), neuroevolution of augmenting topologies (NEAT), vehicle-to-vehicle (V2V) communications

## Abstract

The opportunistic exchange of information between vehicles can significantly contribute to reducing the occurrence of accidents and mitigating their damages. However, in urban environments, especially at intersection scenarios, obstacles such as buildings and walls block the line of sight between the transmitter and receiver, reducing the vehicular communication range and thus harming the performance of road safety applications. Furthermore, the sizes of the surrounding vehicles and weather conditions may affect the communication. This makes communications in urban V2V communication scenarios extremely difficult. Since the late notification of vehicles or incidents can lead to the loss of human lives, this paper focuses on improving urban vehicle-to-vehicle (V2V) communications at intersections by using a transmission scheme able of adapting to the surrounding environment. Therefore, we proposed a neuroevolution of augmenting topologies-based adaptive beamforming scheme to control the radiation pattern of an antenna array and thus mitigate the effects generated by shadowing in urban V2V communication at intersection scenarios. This work considered the IEEE 802.11p standard for the physical layer of the vehicular communication link. The results show that our proposal outperformed the isotropic antenna in terms of the communication range and response time, as well as other traditional machine learning approaches, such as genetic algorithms and mutation strategy-based particle swarm optimization.

## 1. Introduction

Real-time communications between vehicles through vehicular ad hoc networks (VANETs) and intelligent transport systems has been proposed as a possible solution to the increasing number of traffic accidents that has concerned public health officials in recent years [1]. In these systems, key information, such as vehicle speed, acceleration, and position, is periodically transmitted in a two-way manner, in a process known as beaconing, allowing nearing vehicles to be well aware of each other [2].

Vehicle-to-vehicle (V2V) communications is not, however, without problems at intersections [3,4]. In scenarios with too many transmitting vehicles, the channel can get overloaded, and consequently, packets can be lost [5]. One could mitigate this by lowering the rate at which packets are transmitted, but this creates another set of problems, such as widening the discrepancy between the real and estimated position of near cars [6]. Furthermore, shadowing caused by buildings and other objects that obstruct the line of sight (LOS) communication significantly reduces the transmission range, especially at urban intersections [7,8].

The previous problems can be alleviated through beamforming, that is directing the radiation beams in relevant directions, where vehicles can be located [9,10]. In this work, we used beamforming by generating radiation patterns that can overcome obstacles at urban intersections, increasing the range of communication in this type of scenario.

Some research works that have dealt with the presented problems examined the use of roadside units (RSUs) as relay nodes for the communication between the cars [5,8]. However, this solution is too costly, making it impossible to deploy at a large scale. Furthermore, most of the literature regarding RSUs is concerned with the optimization of coverage and cost [11,12], neglecting the minimization of a key aspect in road safety applications: communication delay. Even in works where the position of the RSUs is optimized to decrease the communication delay [13,14], results are not satisfactory regarding road safety applications in dense scenarios.

Unlike RSUs, beamforming does not use relay nodes that can get overloaded in dense scenarios, and thus can provide a communication link with less delay. The main concern in beamforming is finding an algorithm that can calculate the optimal antenna excitation pattern that generates the beam shapes that best adjust to the car’s surroundings.

### 1.1. Related Work

V2V communications rely on the IEEE 802.11p radio access technology [15]. This technology has been specifically designed for the vehicular environment and adopted by the European Telecommunication Standards Institute (ETSI) for supporting the exchange of cooperative awareness messages (CAMs) [2] and decentralized environmental notification messages (DENMs) [16]. On the one hand, CAMs are regularly transmitted by vehicles to provide information about their movement status. The information included in CAMs not only helps vehicles maintain connectivity with their neighbors, but also supports high-level road safety applications. On the other hand, DENMs are generated when a potential risk is detected in order to notify surrounding vehicles about the particular situation. DENMs provide support to event-driven safety applications. Since this work focuses on both the physical layer of V2V communications and safety applications, the default specifications of the IEEE 802.11p standard are adopted, which are a carrier frequency of 5.9 GHz, a transmission rate of 6 Mbps, and a control channel (CCH) of 10 MHz [15].

The problem of shadowing in V2V-intersection scenarios has been usually addressed by using relay nodes [8], such as RSUs, in order to improve the connectivity in critical areas. For instance, References [11,12] focused on covering as much area as possible by deploying a minimum number of RSUs because of the low market penetration of V2V-enabled vehicles and the deployment cost of RSUs. However, the delay permitted for road safety applications was not considered by these works. On the contrary, in [13,14], the RSU placement problem was addressed as an optimization problem in order to minimize the delay, but even so, the values achieved did not satisfy the requirements of road safety applications. In addition, RSUs are not adaptable to changes in their surroundings. This lack of adaptability significantly affects the performance of systems based on RSUs. In this context, the use of beamforming may lower the delay for road safety applications and increase the adaptability to the surroundings.

The problem of using RSUs has been noted, and some investigations have focused on optimizing beam shapes for urban V2V communications. For instance, Reference [17] presented preliminary results on optimizing values for a 4×4 antenna array for urban V2V communications using GA, and it was shown to outperform the baseline isotropic antenna. Similarly, in [18], the values of a 4×4 antenna array were optimized by using particle swarm optimization (PSO) and interpolating the results for unknown positions. This method has also been shown to outperform an isotropic antenna.

### 1.2. Contributions and Organization of the Paper

In this manuscript, we propose a neuroevolution of augmenting topologies (NEAT)-based adaptive beamforming scheme to increase the communication range in V2V-intersection scenarios susceptible to shadowing. Due to NEAT being an unsupervised machine learning technique, its performance is compared with other such algorithms, in particular PSO and genetic algorithm (GA) approaches, as well as to a baseline isotropic antenna. Beamforming is one of the areas where not much is known about the search space, but the objective is clear. Several works have focused on generating and optimizing beam shapes using GAs, showing good results with different types of antenna arrays, e.g., linear [19,20], planar [21,22], T-shaped [23], and 3D arrays [24]. The main goal in [19,20,21,22,23] was to minimize the side lobes’ power, while in [24], the desired beam shape was generated. The specific beam shapes’ generation has been required in mobile communications, in order to adapt to the position of users. Therefore, smart antennas’ control has also been assisted by GA and PSO [25].

In contrast, NEAT uses genetic algorithms to train both the weights and topologies of neural networks. The algorithm was originally proposed to solve the pole balancing problem [26] and has been used in a variety of problems such as controlling robotic arms, learning countermeasures in fighting games, and generating dynamic congestion control algorithms for the transmission control protocol [27,28,29]. The success of NEAT in a variety of problems can be attributed to the capacity the algorithm has to generalize solutions to complex problems.

As can be seen, there is a clear lack of work done in the area of using beamforming in V2V communications to improve communications in urban scenarios, and there are clear reasons to work in this area. In this work, we looked to solve this problem by using algorithms that learn the antenna excitation from its surroundings. In particular, we employed genetic algorithms (GAs) and neuroevolution of augmenting topologies (NEAT). The algorithms were trained and tested in different simulation scenarios focused in urban V2V communications. The main contributions of this manuscript are summarized as follows:

1. We show the positive impact of using evolutionary algorithms with beamforming in urban V2V communication scenarios to learn beam shapes according to the surrounding environment.
2. We propose the use of a neuroevolution algorithm to optimize beam shapes with beamforming for V2V communications in urban scenarios. Unlike other machine learning approaches, such as GA and PSO, NEAT does not require interpolating previously visited positions, since the artificial neural networks take any position as an input.
3. Beamforming with NEAT outperforms the baseline isotropic antenna, as well as beamforming optimized with MSCPSO and GA, in terms of the average response time and the communication range, which are of vital importance for road safety applications.

The remainder of the work is structured as follows. Section 2 presents the details of the models used for the analysis and the evaluations of the proposed schemes. Section 3 describes the optimization algorithms implemented in the work, whereas Section 4 depicts the results and respective analysis. Finally, conclusions are presented in Section 5.

## 2. Methods and Materials

The following section presents details about the channel model and antenna array used for the simulations.

### 2.1. Channel Model

Urban environments are characterized by the presence of various obstacles that deteriorate the performance of V2V communications, especially for vehicles equipped with isotropic antennas in intersection scenarios. To solve this problem, beamforming could be applied to adapt the beam shapes to the context of a specific surrounding environment.

Different algorithms are used to adapt the beam shapes to the surrounding environment. The algorithms are trained through simulations of urban environments. A channel model for urban V2V communications is necessary for the simulations, since this type of environment has many peculiarities that differentiate it from other communication environments. Typically, the channel model presented in [30] is used for urban V2V communications, but [31] presented an improved version of the channel model, which was adopted in this work to estimate the channel realizations. The following equations describe the channel model used for the simulations,

(1) $$G{\left(d_{t},d_{r}\right)}_{dB}=10\;\log_{10}\left(m^{2}\right)+10\;\log_{10}(\underset{\sin\mathrm{gle}\;\mathrm{reflections}}{\underset{︸}{{\left(g_{1}\frac{\lambda }{4\pi (d_{t}+d_{r})}\right)}^{2}}}+\left(\underset{\mathrm{multiple}\;\mathrm{reflections}}{\underset{︸}{g_{2}^{N}\frac{\lambda }{4\pi (d_{t}+d_{r})}{)}^{2}}}\right)+\Psi_{\sigma },$$

where:

(2) $$N=\max\left[2\sqrt{\frac{d_{t}d_{r}}{w_{t}w_{r}}}-1,0\right],$$

**Figure 1:**
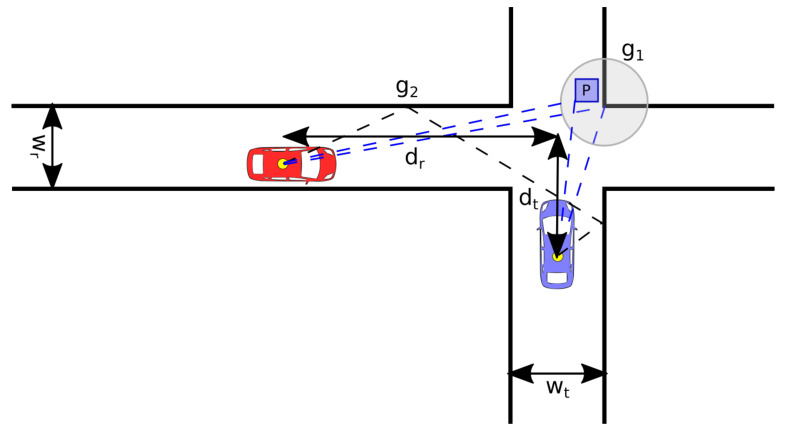
Vehicle-to-vehicle (V2V)-intersection environment.

$d_{t}$ and $d_{r}$ are the distances to the intersection center as shown in Figure 1, *m* is an offset present due to the differences in antenna height, and λ is the wavelength for the transmitted signal. Besides, there are single-order and high-order reflections that contribute to the received power. These reflections are represented by $g_{1}$ and $g_{2}$, where $g_{1}$ is the mean effective amplitude gain from single-reflection interactions and $g_{2}$ is the mean effective amplitude gain from multiple-reflection interactions. $w_{t}$ and $w_{r}$ are the road width for the transmitting and receiving vehicles, respectively; and $\Psi_{\sigma }$ is needed to model the large-scale fading as multiple Gaussian processes, with parameter σ, for each communication link and iteration. Finally, the values $g_{1}$, $g_{2}$, *m*, and σ were estimated from the measurements in [31].

### 2.2. Antenna Array

There are several ways to generate different beam shapes. In the following work, this was achieved by using a uniformly spaced planar array composed of several isotropic antenna. For this type of array, the array factor (AF) is expressed as,

(3) $$AF\left(\theta ,\phi \right)=\sum_{m=0}^{n-1}\sum_{i=0}^{n-1}I_{im}\exp\left(jk\sin\theta (i\Delta x\cos\phi +m\Delta y\sin\phi )\right),$$

where *n* is the number of isotropic antennas on each side of the array. $I_{im}$ is the output power of each antenna in the array, which is a complex value that can be decomposed as $I_{im}=A_{im}e^{j\phi_{im}}$, with amplitude $A_{im}$ and phase $\phi_{im}$ for each of the antennas. Since the array is uniformly spaced, there is a constant separation between antenna elements in each direction, represented by Δx and Δy. θ is the elevation angle perpendicular to the planar array. When an algorithm optimizes the beam shape, $A_{im}$ and $\phi_{im}$ are the parameters that it can control, since the beam shape itself cannot be directly controlled by it.

By using the AF given in Expression (2), the radiation pattern for the antenna array at an angle ϕ and an elevation angle θ, Y(θ,ϕ), can be computed as,

(4) Y(θ,ϕ)=R(θ,ϕ)AF(θ,ϕ).

where R(θ,ϕ) is the radiation pattern of a single antenna in the array. For an isotropic antenna, the value for *R* is uniform across all angles. Using this, the radiation pattern at a given angle is proportional to the value of the AF in the same direction:

(5) Y(θ,ϕ)=AF(θ,ϕ).

During the simulation, the previous expression was used to compute the value for the radiation pattern at a given direction.

## 3. Optimization Methods

The following section exposes the details for the optimization methods. Firstly, the optimization problem is presented. Then, the details about the genetic algorithm and how to interpolate positions that were not used during training are illustrated. The NEAT algorithm is shown and explained in the final section.

### 3.1. Optimization Problem

From Figure 1, we can see that the shape of the beam needs to extend as much as possible in the four directions determined by both streets, in order to maximize, in these directions, the received signal power $P_{i}$, with i∈{1,2,3,4}. Therefore, the optimization problem can be formulated as:

(6) $$P_{i}=\underset{P_{i}}{arg max}\left(\sum_{i=1}^{4}P_{i}\left(A_{j},\phi_{j}\right)-\mu \left(\max_{l}P_{l}\left(A_{j},\phi_{j}\right)-\min_{n}P_{n}\left(A_{j},\phi_{j}\right)\right)\right),$$

where l,n∈{1,2,3,4}:n≠l, and μ is a variable that controls the importance of distributing the power equally. Note that the algorithm must learn to control the values of the amplitude $A_{j}$ and phase $\phi_{j}$ of the jth element of antenna in an array with *k* elements. Consequently, the optimization problem to solve can be reformulated as:

(7) $${\hat{A}}_{j},{\hat{\phi }}_{j}=\underset{\begin{array}{c} A_{j},\phi_{j}\\ \mathrm{subject}\;\mathrm{to}\\ \sum_{m=1}^{k}A_{m}<A \end{array}}{arg max}\left(\sum_{i=1}^{4}P_{i}\left(A_{j},\phi_{j}\right)-\mu \left(\max_{l}P_{l}\left(A_{j},\phi_{j}\right)-\min_{n}P_{n}\left(A_{j},\phi_{j}\right)\right)\right),$$

where ${\hat{A}}_{j}$ and ${\hat{\phi }}_{j}$ are the optimal amplitudes and phases for the array, respectively.

### 3.2. Genetic Algorithm

The main operations related to GA are the creation of the first generation of individuals, crossover, and mutation. In the rest of the section, the selection for each of these operations is presented based on [32].

For the creation of the first generation of individuals, there is no initial guess for the amplitudes or phases, since there is no notion of where the optimal solution may be. The diversity of the initial generation was desired; thus, the amplitude and phase values were obtained from a uniform distribution. However, a constant power for the antenna array was desired; thus, the amplitudes must be normalized by the total power, from which the final values for the amplitudes in the first generation are given by:

(8) $${\tilde{A}}_{j}\left(0\right)=A_{j}\left(0\right)\frac{P^{2}}{\sum_{i=1}^{k}A_{i}{\left(0\right)}^{2}},$$

where ${\tilde{A}}_{j}\left(0\right)$ is the value for the amplitude of the jth antenna obtained in the first generation, $A_{j}\left(0\right)$ is the value for the amplitude of the jth antenna resulting from the first generation before normalization, and *k* is the number of antennas in the array. The antenna array as a whole has a constant output power due to a fixed amount of power being provided to the array by the source. This power is denoted as *P*.

For the crossover operation, the single point crossover [32] was employed over the array of values for the antenna array. The only special consideration when doing the crossover was that the crossover points for the phase and amplitude were used separately; thus, the crossover point for both values may be different. When using crossover schemes, there is a chance that the total power of the antenna array is not *P*; thus, Equation (7) must be used once again to normalize the total power.

For the mutation process, a Gaussian distribution was used for every phase and the amplitude values [33]. This is typically used for mutation when the values are not binary coded, as was the case, since floating values were used. This means that the values for an amplitude and a phase and in each iteration are updated as follows:

(9) $$\phi_{j}^{mut}=\phi_{j}+N\left(0,\sigma_{\phi }\right),$$

(10) $$A_{j}^{mut}=A_{j}+N\left(0,\sigma_{A}\right),$$

where $\phi_{j}^{mut}$ and $A_{j}^{mut}$ are the mutated values for both the phase and amplitude and $\sigma_{\phi }$ and $\sigma_{A}$ are the standard deviation for both values. Once again, the mutation may result in a total power different than *P*; thus, Equation (7) must be used to normalize the total power.

A final consideration is that elitism is used in training, meaning that a small part of the best performing individuals is copied into the next generation unchanged. This process improves performance since the GA does not need to find previously discarded solutions once again.

### 3.3. Interpolation

The GA was used to find the radiation pattern at a given point in the streets. Evidently, it is not possible to calculate the radiation pattern for every position in the streets; thus, a methodology was required to determine the radiation pattern for positions on which the GA had not been trained.

**Figure 2:**
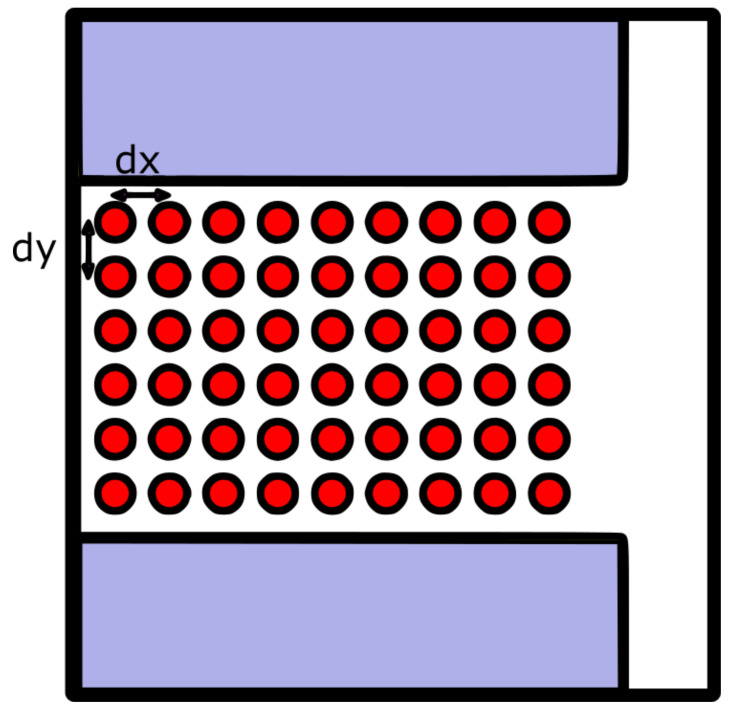
Training and interpolating the genetic algorithm (GA).

The approach used in this work consisted of training the GA on a rectangular grid, with constant values for dx and dy, the separation between points in the grid, and then, interpolating unknown positions from the ones on which the GA had been trained; see Figure 2.

**Figure 3:**
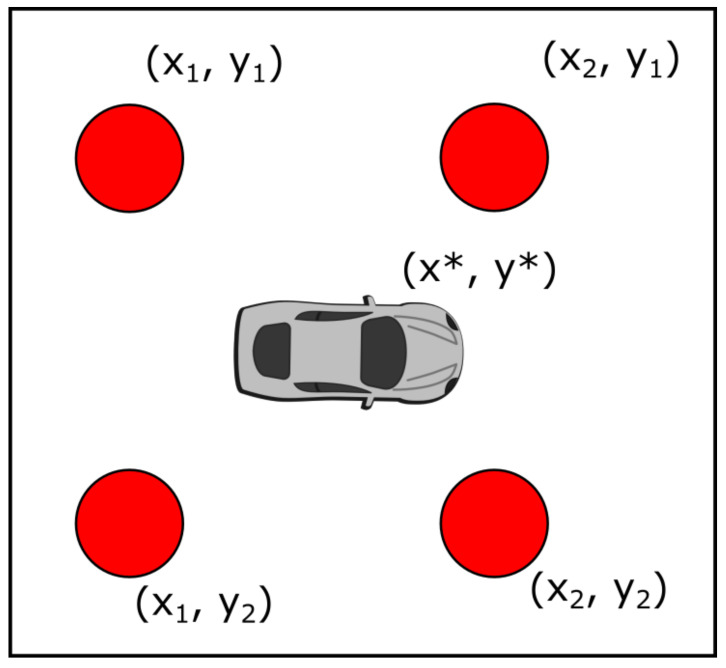
Points used for the interpolation procedure.

In Figure 2, the red circles are the positions at which the GA was trained, which were separated from each other at dx and dy, respectively. To get the radiation pattern for a position $\left(\hat{x},\hat{y}\right)$, on which the GA had not been trained, an interpolation process was used. The radiation pattern for the new position $\left(\hat{x},\hat{y}\right)$, was based on the values of four positions, as can be seen in Figure 3.

Figure 3 is a zoomed-in version of Figure 2, where the car represents the new position $\left(\hat{x},\hat{y}\right)$, and the four red points are the positions used for the interpolation. As can be seen from Figure 3, the four points are at positions $\left(x_{1},y_{1}\right)$, $\left(x_{1},y_{2}\right)$, $\left(x_{2},y_{1}\right)$, and $\left(x_{2},y_{2}\right)$. Point $\left(x_{1},y_{1}\right)$ is denoted by $p_{1}$, $\left(x_{1},y_{2}\right)$ by $p_{2}$, $\left(x_{2},y_{1}\right)$ by $p_{3}$, and $\left(x_{2},y_{2}\right)$ by $p_{4}$. Since these four points were trained with the GA, each of the four points had pairs of values ${\left\{\left(A_{j}^{i},\phi_{j}^{i}\right)\right\}}_{j\in \{1,2,...k\}}$, where *i* denotes the point, thus i∈{1,2,3,4}, and *k* is the number of antenna in the antenna array.

Since the value for the antenna only depends on the values of the amplitude and phase, the antenna of each point can be represented via the following notation:

(11) $$p_{j}^{i}=A_{j}^{i}e^{\phi_{j}^{i}}.$$

With the previous notation, the value of the new position, $\hat{p}$, can be written as a linear combination of the values of $p^{i}$. Thus, the values for $\hat{p}$ results in:

(12) $${\hat{p}}_{j}=\sum_{i=1}^{4}w_{i}p_{j}^{i}.$$

The values for $w_{i}$ should depend on how close the antenna is to the new position. The closer the antenna, the larger the value for $w_{i}$ is. In order to achieve this relationship, the following expressions are employed:

(13) $$w_{i}^{x}=1-\frac{|x\left(p_{i}\right)-\hat{x}|}{x_{1}-x_{0}},$$

(14) $$w_{i}^{y}=1-\frac{|y\left(p_{i}\right)-\hat{y}|}{y_{1}-y_{0}},$$

where $x(p_{i})$ and $y(p_{i})$ are the *x* and *y* positions for the point $p_{i}$, respectively. With this expression, the weights are inversely proportional to the distance between the antenna and the new position. The previous weights are used to calculate $w_{i}$ as $w_{i}=w_{i}^{x}w_{i}^{y}$. Namely, Equation (11) must be written as:

(15) $${\hat{p}}_{j}=\sum_{i=1}^{4}w_{i}^{x}w_{i}^{y}p_{j}^{i},$$

and as mentioned, $p_{i}^{j}$ is given in Equation (10), $w_{i}^{x}$ is given in Equation (12), and $w_{i}^{y}$ is given in Equation (13). A final consideration is that maintaining the same total power for the antenna array regardless of the position was desired because the output power of the antenna should be constant. To this end, the value obtained in Expression (14) must be normalized by a constant total power *P* that is used for every other position. Thus, the final values for the amplitudes denoted ${\tilde{p}}_{j}$ are given by:

(16) $${\tilde{p}}_{j}={\hat{p}}_{j}\frac{P^{2}}{\sum_{i=1}^{k}{\left|{\hat{p}}_{i}\right|}^{2}},$$

which ensures that the total power for the antenna array is *P* regardless of the position.

### 3.4. Neuroevolution of Augmenting Topologies

As previously stated, NEAT relies on training various neural networks through the GA. Due to mixing both algorithms and including different techniques, such as speciation during training, NEAT has a large number of variables. Here, important details about the variables are presented.

The activation function used for the neural network was fixed as the sigmoidfunction, which is the most common activation function in neural networks due to its generalization capability. The bias and weights for each of the layers of the neural network were trained through the GA. Both the bias and weights had minimum and maximum values, which were hyper-parameters, or tunable parameters, for the training/testing model. The rate probability and rate of mutation for the parameters were also the hyper-parameters for the model.

Each neural network started as a fully connected neural network with 2 input neurons, corresponding to the position of the car relative to the center of the intersection, no hidden layers, and 32 output neurons, corresponding to 16 amplitudes and 16 phases for each of the antennas in a 4×4 array.

The fitness for each of the neural networks was given by the value to maximize in Expression (6). The score was computed from one of two scenarios, either by evaluating from a deterministic equidistant distribution of points in the simulation or by evaluating from a random distribution of points in the simulation. This was also considered a hyper-parameter of the model. The probabilities to add or delete nodes and to add or delete connections were also hyper-parameters for the model.

## 4. Results and Discussion

In this section, the results for the work are carefully presented. The section starts by presenting the simulation scenario, as well as the parameters used for the communication channel and the different algorithms. To train the different machine learning algorithms, different positions from the previously presented simulation scenario were used. The output of the antenna array (which was obtained by properly combining each antenna output) for each given position was computed by the algorithm, then the score was computed by using Equation (7), and subsequently, the algorithms were updated in an unsupervised manner. Finally, a carefully evaluation of the results in terms of several metrics is presented.

### 4.1. Simulation Scenario and Parameters

**Figure 4:**
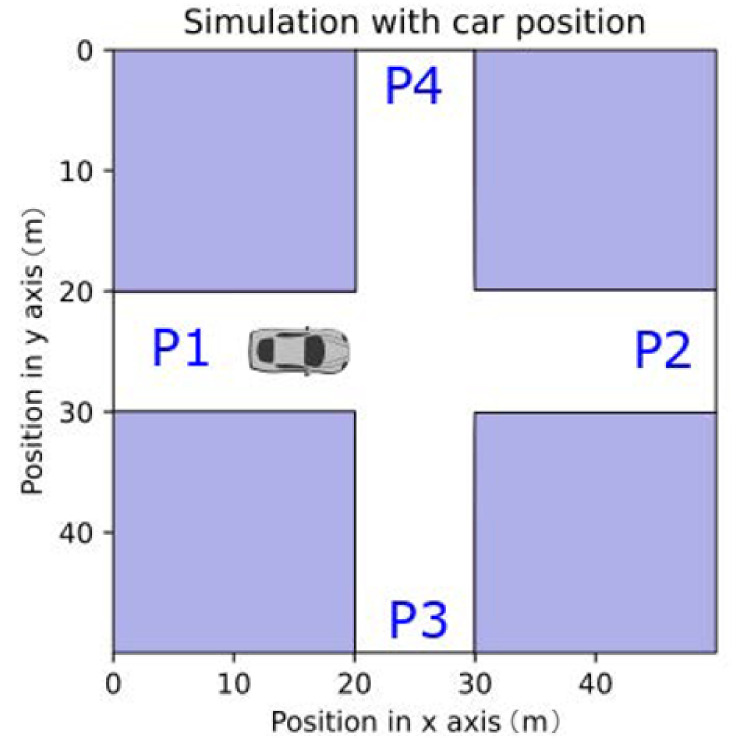
Simulation scenario.

The general simulation scenario is presented in Figure 4. The sections in the white represent the roads in the simulation scenario, and the sections in blue represent the buildings. The car in Figure 4 represents a possible position for the vehicle in the simulation scenario.

Because of the symmetries present in the simulation scenarios, obtaining results from the left side of the horizontal lane was, on average, the same as obtaining results from every position in the simulation scenario. Taking into account this particularity, all the positions for the cars were considered on the left side of the horizontal road.

Furthermore, as the work focused on increasing the communication range for urban V2V communications, the evaluation of the model was done by only considering a single transmitting and receiving vehicle. This simplification had the downside of not taking vehicular density into account. The reason vehicular density was not taken into account was that the work focused on comparing different communication schemes in the same simulation scenario. While taking the vehicular density into account would make the results more accurate, it would affect the communication schemes similarly, and thus, the comparison would be similar. Besides, the complexity of a simulation setup that considers the impact of the vehicular density is much greater, and consequently, the interpretation of the results would also be, so this analysis was outside the scope of this research work.

Nevertheless, the effect of vehicular density in the resulting performance is an interesting point to consider, and thus, it is proposed as future work. To consider this effect, the simulation scenario should be extended to consider multiple vehicles communicating at the same time, and consider the link layer protocols specified in the IEEE 802.11p standard [15]. Interestingly, considering vehicular density might benefit learned beam shapes even further, since models could learn to better utilize the channel by separating it spatially.

As mentioned previously, a channel model for urban V2V communications presented in [31] was used for our observations. This channel model had a number of different parameters that related to the shape of the intersection and were used in Equation (1). Note that the simulation scenario presented in Figure 4 was similar to the X-junction presented in [31]. Consequently, the following parameters were adopted in the work.

The value for the road width presented in Table 1 was calculated by considering the value for the lane width of 4 m presented in [34]. In this work, a road with two lanes and side walks was considered. The side walks had a width of 1 m, which resulted in a road width of 10 m.

As previously noted, this work considered the IEEE 802.11p standard [15] for the physical layer of the vehicular communication link. Table 2 summarizes the key parameters considered for the communication link.

The Rx sensitivity had a slightly higher value than the one presented in [31], which was 23 dBm. This was because the value presented in [31] considered a packet error rate of 10%, which was too high for safety applications in V2V communications. Finally, the algorithms had different hyper-parameters that had to be tuned for successful training, which are illustrated in Table 3.

As mentioned, most of these hyper-parameters were tuned to increase the performance of the respective models. The tuning of the hyper-parameters was done via extensive computer simulations. The training of the model, as well as the performance comparison of the evaluated metrics are presented below.

### 4.2. Performance Evaluation

Taking into account the simulation scenario and the hyper-parameters presented, the following section focuses on presenting and analyzing the results obtained from the simulations.

It is worth mentioning that in [18], MSCPSO was used to optimized the parameters of a 4×4 antenna array using PSO, and the results of the unknown positions were interpolated. However, the latter was evaluated using different restrictions compared to the ones in this manuscript. In particular, the authors in [18] restricted the position of the vehicle in the road, but this restriction was discarded in our manuscript since for safety applications, every possible position is important. New results for MSCPSO were hence computed in this manuscript.

Initially, the results for the average power received in each of the four roads (P1, P2, P3, and P4) are exposed in Table 4. These powers are expressed in dBm.

As can be seen, by using beamforming with any of the algorithms outperformed using an isotropic antenna. The values showed that the performance of the GA and MSCPSO was similar in the vertical roads (P3 and P4), but the GA outperformed MSCPSO significantly in the horizontal roads (P1 and P2). On the other hand, NEAT outperformed both algorithms considerably for all four roads. While the GA and MSCPSO use the interpolation of known positions to generate the output, NEAT trains a neural network that can output the values for any position. This has proven to make NEAT a flexible algorithm that can learn to generalize in various and complex learning tasks.

Another interesting result was the percentage (%) of the positions in which each of the algorithms outperformed the isotropic antenna in each of the four roads. The results were computed by sampling $10^{4}$ positions in order to obtain representative outcomes and comparing the received power for the isotropic antenna and beamforming with each of the algorithms. The results presented in Table 5 show that all of the presented algorithms using beamforming outperformed the isotropic antenna in most scenarios. Even though Table 4 seems to indicate that the performances of the GA and MSCPSO were similar for two of the roads (P3 and P4), Table 5 shows that the GA was superior compared to MSCPSO in all scenarios. This observation seems to indicate that even though, on average, the performance was similar for both algorithms, MSCPSO tended to have much more extreme values when compared to the GA. Table 5 also shows that NEAT outperformed the isotropic antenna in all simulation scenarios. This, once again, was explained by the ability of NEAT to learn general solutions and not relying on the interpolation of known positions.

**Figure 5:**
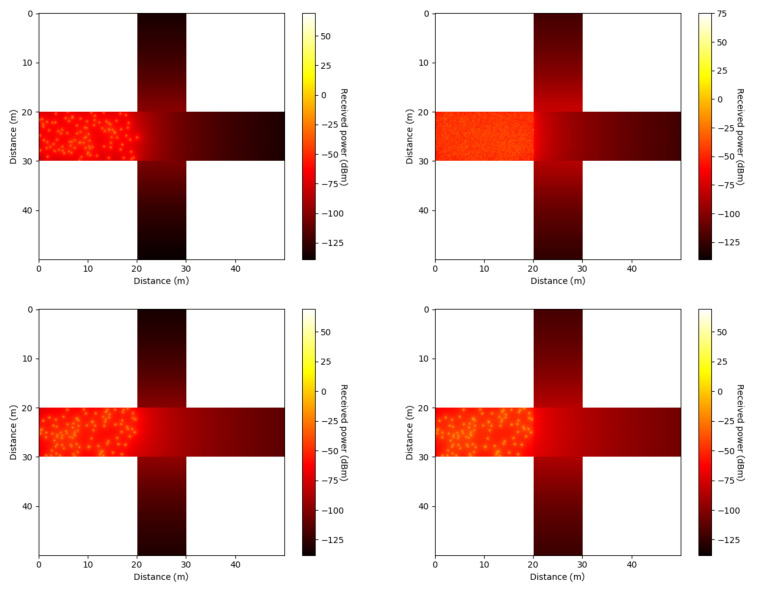
Heat map for the isotropic antenna (**top left**), beamforming with mutation strategy-based particle swarm optimization algorithm (MSCPSO) (**top right**), beamforming with the GA (**bottom left**), and beamforming with neuroevolution of augmenting topologies (NEAT) (**bottom right**).

To expand our results, Figure 5 shows the heat maps of the received power expressed in dBm for the isotropic antenna (top left) and beamforming with MSCPSO (top right), the GA (bottom left), and NEAT (bottom right). The heat-maps visually demonstrate that NEAT had the highest average power reaching the roads, which was reflected in a brighter color in its respective heat map, while the isotropic antenna had the lowest power. The isotropic antenna especially seemed to struggle at the road perpendicular to the car position, which was expected since there was no LOS component and the power was equally distributed in all directions. In comparison with the isometric antenna, both the GA and NEAT improved the power that reached this road by learning to generate beam shapes that focused the power in this specific direction. Meanwhile, MSCPSO depicted significant improvements over the isotropic antenna; however, its results were worse than the GA and NEAT, which was reflected in a darker shade in all four roads overall.

**Figure 6:**
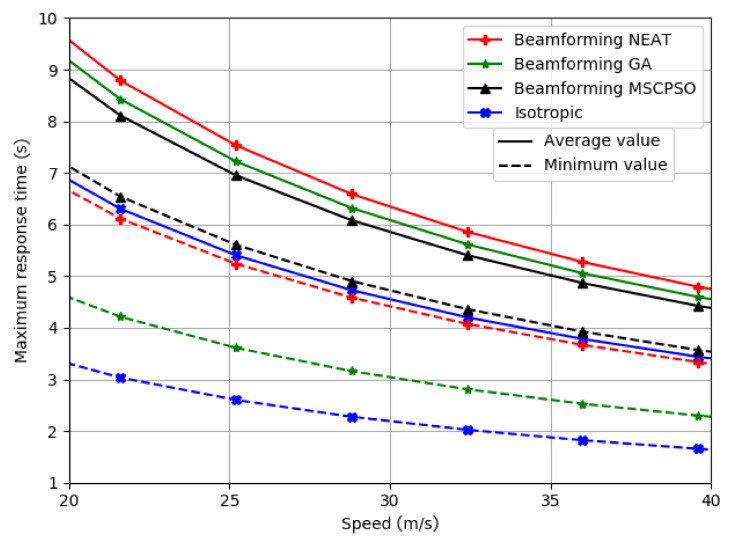
Maximum response time for the different evaluated models.

As mentioned, the main focus of the work was for safety applications. Because of this, one very important result was the amount of time between a vehicle receiving a packet and a possible collision. This was measured by generating $10^{4}$ positions for the car transmitting the packet, by calculating the maximum distance from the intersection where a car moving perpendicular to the transmitting car would receive the packet, and by dividing this distance by the velocity of the car was moving. This relationship is denoted as maximum response time. Figure 6 shows the curves obtained by the procedure that was previously described.

In Figure 6, solid lines represent the mean for the maximum response time calculated for all the positions. The dotted lines represent the value for the worst case scenario. Curves and the respective minimum values for the isotropic antenna and beamforming antenna array optimized with NEAT, the GA, and MSCPSO are presented. It can be seen in Figure 6 that the average maximum response time was largest for NEAT, followed by GA, MSCPSO, and finally, by the isotropic antenna. This shows that, on average, the performance of NEAT was the best, whilst the result for the isotropic antenna was the worst. This is coherent with the results in Table 4 and Table 5, where a similar trend can be appreciated.

It is interesting to note that even though the average value was greater for the GA than for MSCPSO, the GA had a much larger standard deviation, which resulted in a lower minimum value than MSCPSO. It is important to take this into consideration, since for safety applications, this type of border scenario may result in an accident, which was exactly what the work was looking to tackle.

MSCPSO had the highest minimum value for the maximum response time, followed closely by NEAT. This was because MSCPSO had by far the lowest standard deviation for the response time in every position. This means that the algorithm managed to reach long distances at any position in the road, which is important in safety applications. However, NEAT achieved similar minimum values with a slightly larger average value, which indicates that NEAT performed better in most scenarios. Furthermore, both beamforming with NEAT and MSCPSO achieved a minimum performance similar to the average performance of the isotropic antenna. This shows that, even in the worse possible scenario, both algorithms performed similarly to an isotropic antenna on average.

The results show that NEAT and MSCPSO have the best performance for road safety applications, whilst GA and isotropic antenna have much worse performance due to their larger standard deviation and lower average value in the case of isotropic antenna.

## 5. Conclusions

In this article, the use of NEAT along with beamforming was proposed for V2V communications in urban scenarios. The algorithm outperformed other evolutionary algorithms, in particular the GA and MSCPSO, as well as the isotropic antenna in terms of received power and average maximum response time. NEAT also showed similar worse case scenario performance for the maximum response time as MSCPSO, achieving a similar performance to the average values for the isotropic antenna.

The results also showed that NEAT outperformed the isotropic antenna in terms of received power in every position of the simulation. This indicates that using beamforming with NEAT is a net gain in terms of received power, compared to the common isotropic antenna. NEAT also outperformed GA and MSCPSO in terms of average received power, with a difference of over 14 dBm in every direction.

As future work, it would be interesting to try more neuroevolution-based algorithms to further increase the performance of the antenna array. The models could also be tested in real-life scenarios, analyzing the difference in performance between the simulations and real scenarios, while also analyzing the feasibility of employing the different models in real-time urban V2V communications. Furthermore, obtaining results in more realistic scenarios, such as different vehicular densities, is important for real-life applications, and thus proposed as future work.

## Figures and Tables

**Table 1:** Parameters for the simulation scenario.

Parameter	Value
$g_{1}$	0.12 [31]
$g_{2}$	0.58 [31]
*m*	4.18 dB [31]
σ	4.28 dB [31]
Road width	10 m [34]

**Table 2:** Parameters for the communication link.

Parameter	Value
Frequency	5.9 GHz [15]
Data rate	6 Mbps [15]
Beacon rate	10 beacon/s [15]
Tx output power	20 dBm [31]
Rx sensitivity	−67 dBm [31]

**Table 3:** Parameters used for the evaluated algorithms.

Model	Parameter	Value
GA	Individuals	500
	Generations	150
	dx	1 m
	dy	1 m
	$\sigma_{A}^{2}$	10.0
	$\sigma_{\phi }^{2}$	0.5
	Crossover probability	0.8
	Mutation probability	0.3
NEAT	Population size	150
	Generations	300
	Activation function	sigmoid
	Input nodes	2
	Output nodes	32
	Probability to add connection	0.7
	Probability to delete connection	0.3
	Probability to add node	0.4
	Probability to delete node	0.2

**Table 4:** Received power in each road for the isotropic antenna case and beamforming for different algorithms.

Antenna	P1 (dBm)	P2 (dBm)	P3 (dBm)	P4 (dBm)
Isotropic antenna	−112.82	−112.82	−114.16	−124.60
Beamforming with MSCPSO	−105.22	−105.82	−105.07	−110.07
Beamforming with GA [17]	−89.36	−89.30	−108.55	−109.03
Beamforming with NEAT	−74.24	−74.30	−96.35	−95.89

**Table 5:** Percentage of scenarios where the antenna array outperforms the isotropic antenna for different algorithms.

Antenna	P1 (%)	P2 (%)	P3 (%)	P4 (%)
Beamforming with MSCPSO	72.80	69.29	95.25	81.11
Beamforming with GA	99.51	99.48	98.34	96.09
Beamforming with NEAT	100	100	100	100

## Data Availability

Not applicable.

## Abbreviations

The following abbreviations are used in this manuscript:

AF	Array factor
GA	Genetic algorithm
LOS	Line of sight
MSCPSO	Mutation strategy-based particle swarm optimization
NEAT	Neuroevolution of augmenting topologies
PSO	Particle swarm optimization
RSU	Road side unit
VANET	Vehicular ad hoc network
V2V	Vehicle-to-vehicle
